# Anticipation-angle interaction identifies 45° unanticipated change of direction as a high-demand movement with altered ACL injury-related biomechanical characteristics

**DOI:** 10.3389/fbioe.2026.1895455

**Published:** 2026-09-09

**Authors:** Chuangui Mao, Shidong Xie, Ziwen Wang, Xin Yang, Zhiyu Guan, Yuangai Huang, Yongyue Huang, Weiguo Liu

**Affiliations:** College of Physical Education and Health, Guangxi Normal University, Guilin, China

**Keywords:** ACL, biomechanics, change of direction, knee abduction moment, neuromuscular control

## Abstract

Unanticipated change-of-direction (COD) maneuvers represent a key scenario for non-contact lower-limb injury exposure in sport. This study investigated how anticipation modulates lower-limb mechanics across varying cutting angles (45°, 90°, and 180°) to identify phase-specific dynamic joint vulnerabilities. Twenty-three high-level male soccer players performed anticipated (ANT) and unanticipated (UNA) COD tasks. Kinematics, kinetics, and muscle activation were analyzed using two-way repeated-measures ANOVA and Statistical Parametric Mapping (SPM1D). Significant angle effects occurred for temporal and force variables (*p* < 0.05). At 45°, ANT showed higher impact forces and stiffness (*p* < 0.01). Condition × Angle interaction effects emerged for medial gastrocnemius preactivation (*p* = 0.004) and vastus medialis cushion (*p* = 0.007). SPM1D revealed interactions for CoM velocity (*p* < 0.005), early-cushion knee angles (*p* = 0.034), and mid-cushion moments (*p* = 0.024). UNA-45 exhibited greater negative knee power during mid-cushion (*p* < 0.001), while trunk lateral bending interaction occurred during early preactivation (*p* = 0.039). Anticipation effects were maximal at 45°, whereas 90° and 180° mechanics were primarily angle-driven (*p* < 0.001). While 90° and 180° maneuvers provide a temporal buffer for movement reorientation, the 45° task forces athletes to process extreme loads within a compressed timeframe. Under unanticipated conditions, altered feedforward neuromuscular preparation combined with an upright landing posture was accompanied by greater eccentric power absorption, indicating increased multi-planar mechanical demands. These findings suggest that 45° UNA cutting may serve as a challenging biomechanical paradigm for examining biomechanical characteristics relevant to ACL injury mechanisms, revealing potential neuromuscular control adaptations under constrained preparation conditions that may remain less evident during larger-angle or pre-planned maneuvers.

## 1 Introduction

In professional football, unanticipated change of direction (COD) maneuvers are common and may impose mechanical demands associated with anterior cruciate ligament (ACL) injury mechanisms. Recent epidemiological data from European leagues reveal that 77.8% of ACL injuries occur through non-contact mechanisms (Ortiz-Sánchez et al., 2026). Crucially, over 60% of these ruptures happen during off-ball defending, where the requirement to react to unpredictable opponent movements triggers severe biomechanical instability (Della Villa et al., 2020). The professional toll of such injuries is catastrophic: athletes face an average 10.9-month recovery period, a 2.19-fold increase in retirement risk, and nearly 40% of those who return never regain their pre-injury performance peaks (Borque et al., 2024). Such enduring competitive decay indicates that reductionist laboratory assessments often lack ecological validity, as they fail to account for the volatile interplay between cognitive processing and physical mechanics inherent in match-play.

To address these complexities, researchers have progressively unraveled the cognitive mechanisms underlying biomechanical failure during COD. Foundational work established that UNA-induced spikes in knee abduction moments (KAM) are a hallmark of cognitive load, while high cognitive demands were found to generally compromise execution quality (Brown SR et al., 2014; Iguchi et al., 2014). Recent advancements further demonstrate that corrective non-sagittal hip movements used to retrieve center-of-mass stability inadvertently increase peak KAM, severely compressing the planning window and exposing the joint to hazardous loading at initial contact (Sankey et al., 2020).

To fully characterize these movement strategies and mechanical demands, it is essential to consider how cognitive processing time interacts with external physical constraints. On the one hand, anticipation dictates the critical time window available for cognitive processing and neuromuscular motor programming (Sun et al., 2021). Unanticipated execution severely compresses this decision-making window, deprives the motor system of sufficient preparation time, and disrupts feedforward muscle pre-activation before ground contact—a crucial stabilization mechanism given that ACL injury events have been estimated to occur within approximately 17–50 ms after initial contact, a timeframe that precedes effective sensorimotor feedback responses (Bencke and Zebis, 2011; Krosshaug et al., 2007). On the other hand, the cutting angle serves as a primary physical task constraint dictating mechanical requirements. Research into the “angle-velocity trade-off” suggests that sharper directional changes intensify deceleration demands and knee joint loads (Bill et al., 2025; Dos’Santos et al., 2018). This inherently elevates multiplanar kinetic demands, highlighting a fundamental “performance-safety trade-off” where techniques favored for speed—such as stiffer joint strategies and high-impact braking—directly amplify knee joint loading (Dos’Santos et al., 2021). However, despite the established importance of both dimensions, previous studies have often investigated these factors separately or with limited integration (Leppänen et al., 2021). This reductionist approach creates a critical knowledge gap: when elevated mechanical loading demands (sharper angles) collide with a restricted preparation time (unanticipated execution), the kinetic chain’s capacity to safely dissipate forces in the sagittal plane may be compromised, prompting suboptimal non-sagittal compensations and heightened dynamic joint vulnerability—a synergistic interaction that isolated analyses fail to address (Dos’Santos et al., 2021; Maniar et al., 2019).

Investigating the isolated effects of cutting angle or anticipation is insufficient to characterize complex injury mechanisms; therefore, examining their interaction is imperative. Traditional biomechanical approaches typically rely on discrete peak values, which fail to capture the temporal continuum and risk masking transient instabilities throughout the movement phase (Mao et al., 2025). This study uses Statistical Parametric Mapping (SPM1D) to track the kinetic chain’s dynamic evolution throughout the entire stance phase, providing a comprehensive, continuous trajectory analysis to identify critical, phase-specific instability. We hypothesized that a significant interaction exists between anticipation and cutting angle, with unanticipated conditions producing angle-dependent alterations in mechanical loading and neuromuscular control characteristics, particularly when movement regulation is constrained by limited preparation time.

## 2 Materials and methods

### 2.1 Participants

A total of 23 high-level male soccer players (age: 21.76 ± 1.95 years; height: 1.73 ± 0.05 m; body mass: 67.11 ± 6.48 kg; training experience: 9.30 ± 2.05 years) were recruited for this study. Participants were Chinese National First- and Second-Grade soccer players (Rank I: n = 10; Rank II: n = 13). An *a priori* power analysis using G*Power (v3.1.9.7; University of Kiel, Germany) for a 2 × 3 (Condition × Angle) two-way repeated-measures ANOVA (medium effect size *f* = 0.25, α = 0.05, power = 0.80, *r* = 0.5) indicated a minimum sample size of 19 (Dos’Santos et al., 2018; Ebner et al., 2025). Thus, our sample of 23 players was adequately powered to detect a medium-sized interaction effect under the predefined assumptions. Participants were injury-free for the preceding 6 months, refrained from strenuous exercise for 24 h prior to testing, and provided written informed consent. The protocol was approved by the Ethics Committee of Guangxi Normal University (No. 20251029001) in accordance with the Helsinki Declaration.

### 2.2 Experiment procedures

Kinematic data were captured using an 8-camera system (Oqus 700+, Qualisys AB, Göteborg, Sweden) at 200 Hz. Kinetic data were synchronously collected via a force plate (Type 9287CA, 0.6 m × 0.9 m, Kistler Group, Switzerland) at 1,000 Hz. The testing protocol followed a repeated-measures design, with participants performing both anticipated and unanticipated change-of-direction (COD) tasks at angles of 45°, 90°, and 180°. Each session began with a 10-min dynamic warm-up, after which participants performed 20-m maximal sprints to establish baseline velocity. For the COD trials, a 10-m approach was provided to allow sufficient acceleration before the cutting maneuver. Participants were required to achieve an approach velocity of at least 3 m/s prior to directional change, and velocity was monitored using infrared timing gates positioned 3–4 m before the force plate. Only trials meeting both criteria (approach velocity ≥3 m/s and ≥60% of individual maximal sprint velocity) were accepted for further analysis (Alimoradi et al., 2024; Sado et al., 2021; Landry et al., 2007; Besier et al., 2001). To introduce reduced movement preparation demands, the unanticipated condition utilized a custom-built triggering system. When the participant broke an infrared beam positioned 2.5 m before the force plate, a visual LED signal was activated to randomly reveal the target angle. In contrast, the target angle for the anticipated condition was disclosed prior to the start of the approach. For each condition and angle, participants completed three successful trials using their dominant limb—defined as the preferred kicking leg based on self-report—as the plant leg across all subsequent COD tests. A successful trial required valid force-plate contact and completion of the intended COD maneuver without slipping or loss of balance. Trials that did not meet these criteria were repeated until three successful trials were obtained.

To control for potential learning or fatigue effects, the execution order of all conditions and angles was randomized and counterbalanced, with a minimum 48-h intermission between testing sessions. Synchronous with kinematic and kinetic data, surface electromyography (sEMG) signals were recorded at 2,000 Hz using a wireless system (Ultium EMG, Noraxon United States Inc., Scottsdale, AZ). Based on previous literature, seven target muscles of the stance limb were investigated: vastus medialis (VM), vastus lateralis (VL), rectus femoris (RF), semitendinosus (ST), biceps femoris (BF), medial gastrocnemius (MG), and lateral gastrocnemius (LG). Prior to electrode placement, skin preparation was performed in accordance with the SENIAM guidelines. For signal normalization, maximum voluntary isometric contraction (MVIC) tests were conducted immediately following skin preparation. The testing postures strictly adhered to classical literature and SENIAM recommendations: the quadriceps (VM, VL, RF) were tested during resisted knee extension in a seated position with 70°–90° of knee flexion; the hamstrings (ST, BF) were evaluated during resisted knee flexion in a prone position with 20°–30° of knee flexion; and the gastrocnemius (MG, LG) was assessed during resisted plantarflexion in a 90° ankle position (Besomi et al., 2020). To ensure true maximal neural drive and compliance, real-time visual feedback of the sEMG was continuously displayed on a screen, supplemented by standardized verbal encouragement (e.g., rhythmic, high-intensity vocal prompts) from the same investigator across all MVIC trials.

### 2.3 Data processing

To minimize intra-subject variability, data from these three successful trials were trial-averaged within each participant prior to subsequent statistical analysis. A modified Helen Hayes marker set was used to construct the Visual3D biomechanical model, including lower-extremity segments and a thorax segment for trunk kinematic analysis. Segment inertial properties for inverse-dynamics calculations were assigned using the default Visual3D segment parameter settings without additional modification, and the same settings were applied across all participants. Kinematic and kinetic data were filtered using fourth-order zero-lag Butterworth low-pass filters at cutoff frequencies of 14 Hz and 50 Hz, respectively (Mao et al., 2025). Joint kinematics and kinetics were computed using Visual3D (v6, C-Motion Inc., Germantown, MD, United States) based on Joint Coordinate System conventions with the right-hand rule. Trunk kinematics were derived from the thorax segment of the biomechanical model. Positive values defined flexion, lateral bending, and axial rotation for the trunk, as well as extension, abduction, internal rotation, and power generation for the knee. The movement was divided into three phases (Ortiz-Sánchez et al., 2026): preactivation (the standardized 150-ms interval preceding initial contact [IC]) (DeMont et al., 2026; Bai et al., 2019; Della Villa et al., 2020) cushion (from IC to peak knee flexion); and (Borque et al., 2024) extension (from peak knee flexion to toe-off). IC and toe-off were identified using a 20 N ground reaction force (GRF) threshold (Iguchi et al., 2014; Sado et al., 2021). COD speed was defined as the average resultant velocity of the center of mass (CoM) in the horizontal plane across each phase, calculated frame-by-frame using Equation 1:

$$v_{resultant}=\sqrt{v_{AP}^{2}{+v}_{ML}^{2}}$$ (1)

where $v_{AP}\text{ and }v_{ML}$ represent the anteroposterior and mediolateral CoM velocity components, respectively. All joint moments, powers, and mechanical work were computed as net internal entities in Visual3D. Key kinetic metrics—including peak vertical GRF, net internal knee moments, and joint work—were extracted and normalized to body mass (N/kg for GRF, Nm/kg for moments, and J/kg for work, respectively) (Dos’Santos et al., 2018; Sado et al., 2021). Knee joint power in each plane was calculated as the product of the net internal knee joint moment (*M*_*interna*l_) and the joint angular velocity (ω), as shown in Equation 2:

$$P=M_{\text{internal}}\cdot \;\omega$$ (2)

Mechanical joint work (*W*) during specific phases was subsequently determined by integrating the joint power curve over time, as shown in Equation 3:

W=∫P dt (3)

Knee joint stiffness (*K*, Nm/kg) in the sagittal plane was determined to reflect the limb’s resistance to deformation (Liew et al., 2021), and was calculated as the change in the net internal knee flexion/extension moment (ΔM) divided by the change in the knee flexion/extension angle (Δθ), as shown in Equation 4:

$$K=\frac{\Delta M}{\Delta \theta }$$ (4)

For sEMG data processing, raw signals were band-pass filtered between 10 and 500 Hz using a fourth-order zero-lag Butterworth filter, full-wave rectified, and low-pass filtered at 6 Hz (Zebis et al., 2009). RMS amplitudes were then calculated from the processed EMG signals separately within the three predefined movement phases (preactivation, cushion, and extension). RMS amplitudes were normalized to the maximal RMS value obtained during MVIC trials and expressed as %MVC. Specifically, the RMS value calculated for each movement phase was divided by the corresponding MVIC reference value. During each 5-s MVIC trial, the highest 3-s RMS value was selected as the normalization reference. Trials with evident EMG artifacts or poor signal quality were excluded from subsequent EMG analysis. For frequency-domain analysis, MDF was computed across all phases in MATLAB using the Welch method with a segment length of 128 samples on the filtered, non-rectified signals (Bill et al., 2025; Besier et al., 2003; Jones et al., 2016).

### 2.4 Statistical analysis

Based on the study hypothesis, variables related to joint loading and neuromuscular control were considered primary outcomes, whereas additional variables were analyzed to provide complementary information on movement strategies. Discrete variables and time-series trajectories were analyzed using SPSS 26.0 and SPM1D (v.0.4) in MATLAB, respectively (Mao et al., 2025). Time-series waveforms were time-normalized to 101 data points (0%–100%) via linear interpolation, and averaged across the three successful trials for each participant per condition to yield subject-level mean curves prior to SPM analysis. Normality and sphericity were assessed, with Greenhouse-Geisser corrections applied as needed. A 2 × 3 (Condition × Angle) repeated-measures ANOVA (α = 0.05) was used to assess main effects and interactions, and the interaction was emphasized according to the study objective. Significant main effects and interactions were further decomposed using post-hoc pairwise comparisons. Bonferroni corrections were applied for comparisons among cutting angles (45°, 90°, and 180°) to adjust for multiple comparisons (adjusted α = 0.017). For discrete outcomes, multiplicity control was prespecified and applied within each predefined dependent-variable statistical model. Bonferroni adjustment was applied only to post-hoc pairwise comparisons following significant ANOVA effects, rather than across unrelated biomechanical outcome domains. For time-series trajectories, SPM1D identified significant clusters where the F statistic exceeded the Random Field Theory-based threshold, controlling continuum-level family-wise error at α = 0.05 (Pataky et al., 2015). Effect sizes were reported as partial eta squared ( $\eta_{p}^{2}$ ) for ANOVA (<0.01 negligible; 0.01–0.06 small; 0.06–0.14 medium; ≥0.14 large) and Cohen’s d for pairwise comparisons.

## 3 Results

Results are presented with emphasis on biomechanical variables directly related to joint loading and neuromuscular control, while additional outcomes are reported to provide complementary characterization of COD strategies. As summarized in Table 1, the two-way repeated-measures ANOVA revealed a highly significant main effect of angle for all temporal variables (*p* < 0.001, $\eta_{p}^{2}$ > 0.682), showing that the durations of the cushion, extension, and total support phases increased significantly with the cutting angle (45° < 90° < 180°, all *p* < 0.010). A highly significant main effect of angle was also found for the cushion phase (*F*_(2,44)_ = 68.551, *p* < 0.001, $\eta_{p}^{2}$ = 0.682), extension phase (*F*_(2,44)_ = 116.176, *p* < 0.001, $\eta_{p}^{2}$ = 0.784), and total support phase (*F*_(2,44)_ = 30.309, *p* < 0.001, $\eta_{p}^{2}$ = 0.897). Similarly, a highly significant main effect of angle was observed for both peak impact force (*F*_(2,44)_ = 136.878, *p* < 0.001, $\eta_{p}^{2}$ = 0.811) and peak propulsive force (*F*_(2,44)_ = 134.707, *p* < 0.001, $\eta_{p}^{2}$ = 0.808). No significant main effects of anticipation or interaction effects were observed for either variable.

**TABLE 1:** Temporal variables and peak ground reaction forces (N = 23).

Variables	Con.	Angle (mean ± SD)			ANOVA					
					Anticipation		Angle		Interaction	
		45°	90°	180°	*p*	$\eta_{p}^{2}$	*p*	$\eta_{p}^{2}$	*p*	$\eta_{p}^{2}$
Cushion phase (s)	ANT	0.08 ± 0.02^aa^	0.11 ± 0.04^bb^	0.17 ± 0.06^cc^	0.215	0.048	**<0.001**	**0.682**	0.546	0.014
	UNA	0.09 ± 0.03^aa^	0.12 ± 0.04^bb^	0.19 ± 0.05^cc^						
Extension phase (s)	ANT	0.12 ± 0.02_aa_	0.16 ± 0.03^bb^	0.29 ± 0.08^cc^	0.082	0.091	**<0.001**	**0.784**	0.171	0.057
	UNA	0.14 ± 0.02^aa^	0.16 ± 0.03^bb^	0.34 ± 0.10^cc^						
Support phase (s)	ANT	0.20 ± 0.03^aa^	0.27 ± 0.06^bb^	0.46 ± 0.08^cc^	0.050	0.115	**<0.001**	**0.897**	0.050	0.100
	UNA	0.22 ± 0.04^aa^	0.28 ± 0.06^bb^	0.53 ± 0.10^cc^						
Peak impact force (N/kg)	ANT	30.40 ± 4.97	28.85 ± 7.27^bb^	20.63 ± 4.80^cc^	0.075	0.096	**<0.001**	**0.811**	0.592	0.016
	UNA	25.47 ± 3.91	27.69 ± 5.83^bb^	23.14 ± 6.73						
Peak propulsive force (N/kg)	ANT	24.20 ± 2.45^aa^	21.32 ± 2.72^bb^	16.20 ± 1.57_cc_	0.253	0.041	**<0.001**	**0.808**	0.573	0.017
	UNA	22.77 ± 2.14^aa^	20.34 ± 2.40^bb^	15.72 ± 1.50^cc^						

ANT, anticipated condition; UNA, unanticipated condition. a/b/c: angle pairwise differences (45°/90°, 90°/180°, 45°/180°). : ANT vs. UNA effect at same angle. Bold values indicate statistically significant effects (*p* < 0.05). Single/double symbols: *p* < 0.05/*p* < 0.01.

As presented in Table 2, within the knee joint kinetics domain, a significant main effect of angle was captured for knee flexion work during the cushion phase (*F*_(2, 44)_ = 12.242, *p* < 0.001, $\eta_{p}^{2}$ = 0.277). Similarly, a significant main effect of angle was observed for abduction work during the cushion phase (*F*_(2, 44)_ = 9.837, *p* < 0.001, $\eta_{p}^{2}$ = 0.235). A significant main effect of angle was also found for rotation work during the cushion phase (*F*_(2,44)_ = 4.853, *p* = 0.011, $\eta_{p}^{2}$ = 0.132), although post-hoc comparisons revealed no significant pairwise differences between specific angles. However, during the extension phase, a highly significant main effect of angle was identified for flexion stiffness (*F*_(2, 44)_ = 29.276, *p* < 0.001, $\eta_{p}^{2}$ = 0.478). Regarding neuromuscular control outcomes, statistical analysis of muscle activation revealed widespread angular modulations alongside distinct interaction effects across different movement phases. During the preactivation (PRE) phase, significant main effects of angle were observed for the majority of muscles, including the medial gastrocnemius (*F*_(2, 44)_ = 8.399, *p* = 0.001, $\eta_{p}^{2}$ = 0.208), lateral gastrocnemius (*F*_(2, 44)_ = 6.493, *p* = 0.003, $\eta_{p}^{2}$ = 0.169), vastus medialis (*F*_(2, 44)_ = 7.153, *p* = 0.002, $\eta_{p}^{2}$ = 0.183), biceps femoris (*F*_(2, 44)_ = 7.799, *p* < 0.001, $\eta_{p}^{2}$ = 0.271), and semitendinosus (*F*_(1.54, 33.88)_ = 11.181, *p* < 0.001, $\eta_{p}^{2}$ = 0.259). Crucially, a highly significant Condition × Angle interaction effect was captured exclusively for the MG during this phase (*F*_(2, 44)_ = 5.949, *p* = 0.004, $\eta_{p}^{2}$ = 0.157). Subsequent post-hoc simple effect analysis for the MG interaction revealed that within the 180° cutting angle, the UNA condition elicited a significantly higher RMS amplitude than the ANT condition (*p* < 0.01). Throughout the subsequent cushion (CSH) phase, the main effect of angle was thoroughly documented across almost all lower limb muscles. Highly significant angular modulations were detected for the MG (*F*_(2, 44)_ = 33.457, *p* < 0.001, $\eta_{p}^{2}$ = 0.511), LG (*F*_(2, 44)_ = 34.939, *p* < 0.001, $\eta_{p}^{2}$ = 0.522), VM (*F*_(2, 44)_ = 90.656, *p* < 0.001, $\eta_{p}^{2}$ = 0.739), vastus lateralis (VL) (*F*_(2, 44)_ = 63.739, *p* < 0.001, $\eta_{p}^{2}$ = 0.666), rectus femoris (RF) (*F*_(1.54, 33.88)_ = 22.236, *p* < 0.001, $\eta_{p}^{2}$ = 0.410), and BF (*F*_(2, 44)_ = 9.799, *p* < 0.001, $\eta_{p}^{2}$ = 0.234). The ST also exhibited a significant but less pronounced main effect of angle (*F*_(2, 44)_ = 3.516, *p* = 0.036, $\eta_{p}^{2}$ = 0.099). Notably, a highly significant Condition × Angle interaction emerged for the VM during this phase (*F*_(2, 44)_ = 5.387, *p* = 0.007, $\eta_{p}^{2}$ = 0.144), where further pairwise decomposition indicated that VM activation was significantly lower in the UNA condition compared to the ANT condition specifically at the 180° angle (*p* < 0.05). Conversely, during the final extension (EXT) phase, the prominence of angular modulation largely subsided. A significant main effect of angle persisted only for the LG (*F*_(2, 44)_ = 3.358, *p* = 0.041, $\eta_{p}^{2}$ = 0.095) and VM (*F*_(2, 44)_ = 10.189, *p* < 0.001, $\eta_{p}^{2}$ = 0.242). No significant main effects of anticipation or interaction effects were detected for any other muscle variables during this phase (*p* > 0.05).

**TABLE 2:** Analysis of knee joint kinetics and muscle RMS (N = 23).

Variables	Phase	Con.	Angle (mean ± SD)			ANOVA					
						Anticipation		Angle		Interaction	
			45°	90°	180°	*p*	$\eta_{p}^{2}$	*p*	$\eta_{p}^{2}$	*p*	$\eta_{p}^{2}$
I. Knee joint kinetics											
Flexion work (J/kg)	CSH	ANT	−0.57 ± 0.57^aa^	−0.95 ± 0.55	−0.75 ± 0.40	0.426	0.020	**<0.001**	**0.277**	0.226	0.045
		UNA	−0.33 ± 0.42^aa^	−0.79 ± 0.63	−0.81 ± 0.38^cc^						
	EXT	ANT	0.59 ± 0.28	0.63 ± 0.35	0.64 ± 0.45	0.738	0.004	0.863	0.005	0.964	0.001
		UNA	0.64 ± 0.34	0.65 ± 0.35	0.67 ± 0.42						
Abduction work (J/kg)	CSH	ANT	−0.00 ± 0.06	−0.03 ± 0.13	−0.07 ± 0.10	0.664	0.006	**<0.001**	**0.235**	0.650	0.013
		UNA	−0.00 ± 0.03	−0.04 ± 0.09	−0.10 ± 0.13^cc^						
	EXT	ANT	0.06 ± 0.05	0.05 ± 0.08	0.05 ± 0.10	0.613	0.008	0.430	0.026	0.261	0.041
		UNA	0.05 ± 0.06	0.08 ± 0.08	0.07 ± 0.11						
Rotation work (J/kg)	CSH	ANT	−0.01 ± 0.07	−0.03 ± 0.08	0.01 ± 0.06	0.672	0.006	**0.011**	**0.132**	0.720	0.010
		UNA	0.00 ± 0.03	−0.03 ± 0.07	0.01 ± 0.06						
	EXT	ANT	0.01 ± 0.01	0.01 ± 0.03	0.01 ± 0.02	0.259	0.040	0.345	0.033	0.738	0.009
		UNA	0.01 ± 0.01	0.01 ± 0.02	0.02 ± 0.02						
Flexion stiffness (nm/°/kg)	CSH	ANT	1.14 ± 3.78	0.15 ± 2.12	0.05 ± 0.03	0.333	0.029	0.186	0.051	0.408	0.028
		UNA	0.25 ± 2.46	0.12 ± 2.07	0.05 ± 0.03						
	EXT	ANT	0.08 ± 0.02	0.08 ± 0.03^bb^	0.04 ± 0.03^cc^	0.072	0.098	**<0.001**	**0.478**	0.409	0.028
		UNA	0.06 ± 0.02	0.07 ± 0.03^bb^	0.04 ± 0.03^cc^						
II. Muscle activation (RMS)											
Medial gastrocnemius (%)	PRE	ANT	0.32 ± 0.14	0.35 ± 0.16	0.33 ± 0.08	0.428	0.020	**0.001**	**0.208**	**0.004**	**0.157**
		UNA	0.28 ± 0.14	0.37 ± 0.16	0.44 ± 0.17						
	CSH	ANT	0.79 ± 0.19^aa^	0.74 ± 0.16^bb^	0.53 ± 0.20^cc^	0.806	0.002	**<0.001**	**0.511**	0.909	0.003
		UNA	0.77 ± 0.13^aa^	0.72 ± 0.17^bb^	0.54 ± 0.14_cc_						
	EXT	ANT	0.60 ± 0.12	0.55 ± 0.16	0.52 ± 0.14	0.802	0.002	0.102	0.069	0.803	0.007
		UNA	0.58 ± 0.14	0.57 ± 0.13	0.53 ± 0.16						
Lateral gastrocnemius (%)	PRE	ANT	0.26 ± 0.15^aa^	0.28 ± 0.18^bb^	0.36 ± 0.18^cc^	0.998	0.001	**0.003**	**0.169**	0.319	0.035
		UNA	0.26 ± 0.12^aa^	0.28 ± 0.14^bb^	0.36 ± 0.16^cc^						
	CSH	ANT	0.79 ± 0.18^aa^	0.71 ± 0.14^bb^	0.54 ± 0.20^cc^	0.360	0.026	**<0.001**	**0.522**	0.281	0.039
		UNA	0.74 ± 0.13^aa^	0.67 ± 0.11^bb^	0.49 ± 0.13^cc^						
	EXT	ANT	0.61 ± 0.18^a^	0.56 ± 0.23^b^	0.54 ± 0.17^c^	0.906	0.001	**0.041**	**0.095**	0.519	0.020
		UNA	0.60 ± 0.15^a^	0.57 ± 0.16^b^	0.53 ± 0.14^c^						
Vastus medialis (%)	PRE	ANT	0.44 ± 0.13^a^	0.42 ± 0.14^b^	0.32 ± 0.13^c^	0.375	0.025	**0.002**	**0.183**	0.191	0.050
		UNA	0.40 ± 0.14^a^	0.38 ± 0.14^b^	0.36 ± 0.15^c^						
	CSH	ANT	0.89 ± 0.04^aa^	0.84 ± 0.09^bb^	0.64 ± 0.17^cc^	0.392	0.023	**<0.001**	**0.739**	**0.007**	**0.144**
		UNA	0.88 ± 0.05^aa^	0.81 ± 0.11^bb^	0.56 ± 0.13^cc^						
	EXT	ANT	0.44 ± 0.10^aa^	0.46 ± 0.11^bb^	0.58 ± 0.09^cc^	0.593	0.009	**<0.001**	**0.242**	0.986	0.001
		UNA	0.47 ± 0.13^aa^	0.49 ± 0.14^bb^	0.56 ± 0.10^cc^						
Vastus lateralis (%)	PRE	ANT	0.39 ± 0.14	0.34 ± 0.16	0.34 ± 0.17	0.574	0.010	0.748	0.009	0.441	0.025
		UNA	0.36 ± 0.12	0.37 ± 0.14	0.37 ± 0.14						
	CSH	ANT	0.88 ± 0.05^aa^	0.78 ± 0.11^bb^	0.61 ± 0.19^cc^	0.431	0.019	**<0.001**	**0.666**	0.634	0.014
		UNA	0.86 ± 0.09^aa^	0.75 ± 0.12^bb^	0.56 ± 0.14^cc^						
	EXT	ANT	0.49 ± 0.16	0.46 ± 0.20	0.49 ± 0.18	0.338	0.029	0.449	0.025	0.776	0.008
		UNA	0.45 ± 0.13	0.40 ± 0.14	0.45 ± 0.17						
Rectus femoris (%)	PRE	ANT	0.36 ± 0.18	0.36 ± 0.15	0.40 ± 0.17	0.350	0.027	0.088	0.073	0.984	0.001
		UNA	0.31 ± 0.14	0.31 ± 0.14	0.40 ± 0.16						
	CSH	ANT	0.77 ± 0.16^aa^	0.74 ± 0.14^bb^	0.54 ± 0.18^cc^	0.371	0.025	**<0.001**	**0.410**	0.949	0.002
		UNA	0.72 ± 0.18^aa^	0.70 ± 0.10^bb^	0.51 ± 0.12^cc^						
	EXT	ANT	0.44 ± 0.14	0.39 ± 0.18	0.43 ± 0.16	0.947	0.001	0.147	0.058	0.454	0.024
		UNA	0.44 ± 0.12	0.38 ± 0.16	0.43 ± 1.15						
Biceps femoris (%)	PRE	ANT	0.61 ± 0.16^a^	0.62 ± 0.16^b^	0.52 ± 0.16^c^	0.713	0.004	**<0.001**	**0.271**	0.884	0.004
		UNA	0.60 ± 0.12^a^	0.60 ± 0.12^b^	0.49 ± 0.14^c^						
	CSH	ANT	0.73 ± 0.20	0.76 ± 0.16	0.60 ± 0.20	0.914	0.001	**<0.001**	**0.234**	0.211	0.047
		UNA	0.72 ± 0.15	0.75 ± 0.13	0.61 ± 0.13						
	EXT	ANT	0.47 ± 0.15	0.44 ± 0.14	0.52 ± 0.14	0.762	0.003	0.056	0.086	0.949	0.002
		UNA	0.45 ± 0.12	0.43 ± 0.13	0.51 ± 0.16						
Semitendinosus (%)	PRE	ANT	0.67 ± 0.14^a^	0.63 ± 0.17^b^	0.51 ± 0.17^c^	0.782	0.002	**<0.001**	**0.259**	0.188	0.051
		UNA	0.66 ± 0.12^a^	0.59 ± 0.13^b^	0.57 ± 0.16^c^						
	CSH	ANT	0.69 ± 0.18^a^	0.71 ± 0.14^b^	0.63 ± 0.20^c^	0.996	0.001	**0.036**	**0.099**	0.776	0.008
		UNA	0.69 ± 0.17^a^	0.71 ± 0.11^b^	0.63 ± 0.16^c^						
	EXT	ANT	0.50 ± 0.18	0.45 ± 0.16	0.48 ± 0.18	0.071	0.098	0.528	0.020	0.126	0.063
		UNA	0.43 ± 0.15	0.40 ± 0.11	0.42 ± 0.13						

ANT, anticipated condition; UNA, unanticipated condition. PRE, preactivation; CSH, cushion; EXT, extension. a/b/c: angle pairwise differences (45°/90°, 90°/180°, 45°/180°). : ANT vs. UNA effect at same angle. Bold values indicate statistically significant effects (*p* < 0.05). Single/double symbols: *p* < 0.05/*p* < 0.01.

Regarding the frequency domain, no significant main effects or interaction effects were observed for the Median Frequency (MDF) across all analyzed muscles and movement phases (all *F* ≤ 2.169, *p* > 0.05; see Supplementary Table A1 for detailed statistical outputs).

**FIGURE 1:**
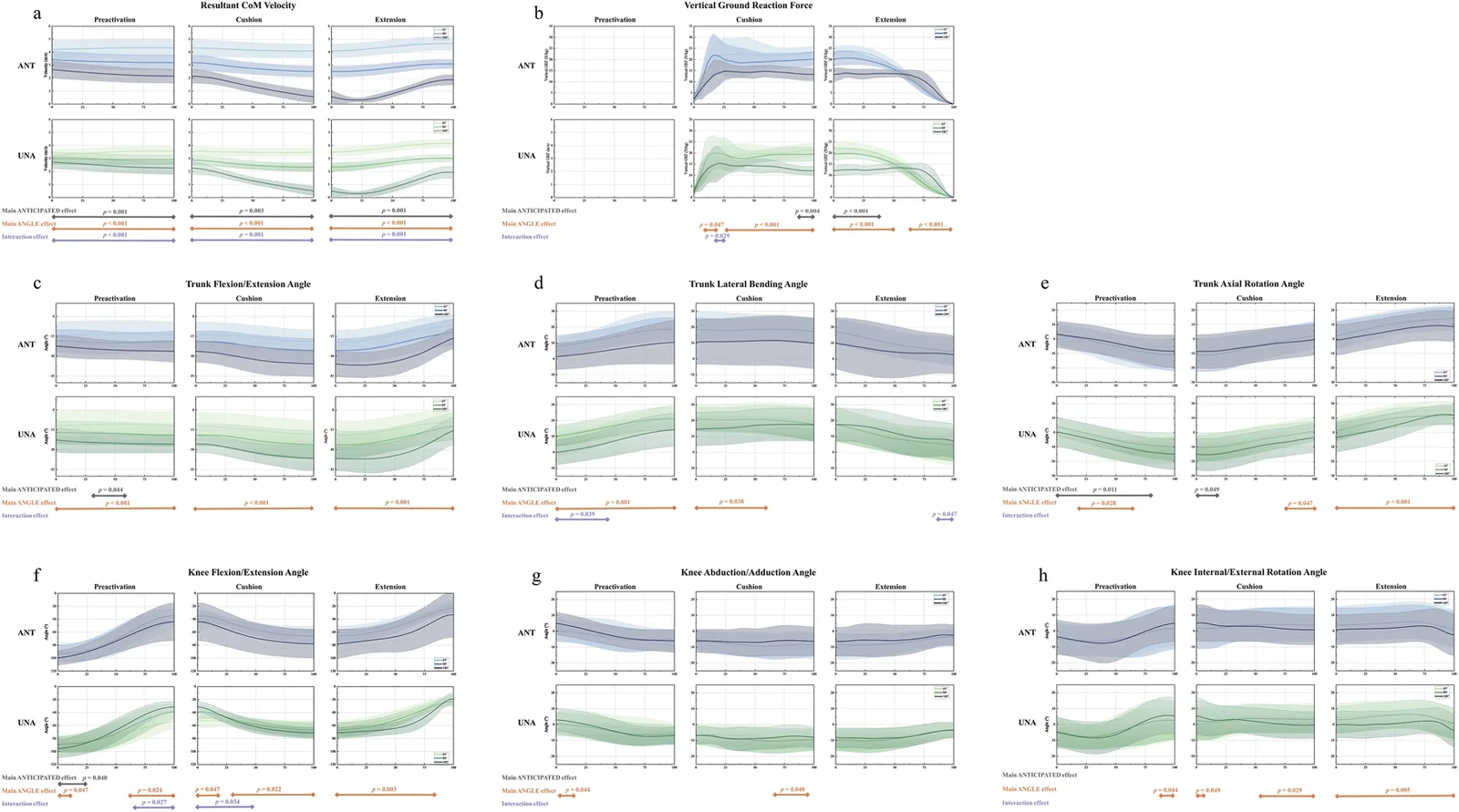
Resultant CoM velocity, vertical GRF, and joint angle profiles. **(a)** Resultant CoM Velocity; **(b)** Vertical Ground Reaction Force; **(c)** Trunk Flexion/Extension Angle; **(d)** Trunk Lateral Bending Angle; **(e)** Trunk Axial Rotation Angle; **(f)** Knee Flexion/Extension Angle; **(g)** Knee Abduction/Adduction Angle; **(h)** Knee Internal/External Rotation Angle. Mean (solid line) and standard deviation (shaded area) of variables are presented for Anticipated (ANT, blue) and Unanticipated (UNA, green) conditions at three cutting angles (45°, 90°, and 180°). The movement is divided into three phases: Preactivation (−150 m to initial contact), Cushion (initial contact to maximum knee flexion), and Extension (maximum knee flexion to toe-off). Horizontal bars below the curves indicate periods of significant effects identified by SPM1D analysis (*p* < 0.05): grey bars for the main effect of Anticipation, orange bars for the main effect of Angle, and purple bars for the Interaction effect. Positive values indicate trunk flexion, lateral bending, axial rotation, and knee extension, abduction, internal rotation.

As a primary indicator of movement regulation under altered preparation demands, resultant CoM velocity exhibited significant main effects of anticipation and angle (Figure 1a), as well as a significant interaction, throughout the preactivation (0.0%*–*100.0%, *p <* 0.001), cushion (0.0%*–*100.0%, *p <* 0.005), and extension phases (0.0%*–*100.0%, *p =* 0.001). As illustrated in Figure 1b, vertical GRF showed significant main effects for anticipation (82.8%*–*100.0%, *p =* 0.004), angle (8.0%*–*19.1%, *p =* 0.018; 26.2%*–*100.0%, *p <* 0.001), and interaction (18.4%*–*26.7%, *p =* 0.029) during the cushion phase, while the extension phase exhibited significant anticipation (0.0%*–*37.6%, *p <* 0.001) and angle effects (0.0%*–*52.9%, *p <* 0.001; 64.8%*–*99.9%, *p <* 0.001). Trunk flexion/extension angles exhibited a significant anticipation effect during preactivation (31.1%*–*60.7%, *p =* 0.044) and significant angle effects across all three phases (0.0%*–*100.0%, *p <* 0.001) as shown in Figure 1c. Trunk lateral bending angles showed significant angle effects during preactivation (0.0%*–*100.0%, *p <* 0.001) and cushion (0.0%*–*59.8%, *p =* 0.038), with interactions identified during early preactivation (0.0%*–*40.4%, *p =* 0.039) and late extension (82.3%*–*100.0%, *p =* 0.047) as shown in Figure 1d. Trunk axial rotation was significant for anticipation (0.0%*–*79.7%, *p =* 0.011) and angle (16.2%*–*65.1%, *p =* 0.028) during preactivation as shown in Figure 1e. During the cushion phase, anticipation (0.0%*–*16.2%, *p =* 0.049) and angle effects (75.9%*–*100.0%, *p =* 0.047) were significant, followed by a continuous angle effect throughout extension (0.0%*–*100.0%, *p <* 0.001). Knee flexion/extension angles were significantly affected during the cushion phase by angle effects (0.0%*–*18.3%, *p =* 0.047; 30.1%*–*100.0%, *p =* 0.022) and an early interaction (0.0%*–*48.0%, *p =* 0.034) were significant as shown in Figure 1f. A sustained angle effect was observed during the extension phase (0.0%*–*85.7%, *p =* 0.003). Knee abduction/adduction angles exhibited significant angle effects during late cushion (66.4%*–*95.3%, *p =* 0.040; Figure 1g). Internal/external rotation angles showed significant angle effects during cushion (0.0%*–*9.1%, *p =* 0.049; 55.3%*–*100.0%, *p =* 0.029), and throughout the extension phase (0.0%*–*100.0%, *p =* 0.005) as shown in Figure 1h.

**FIGURE 2:**
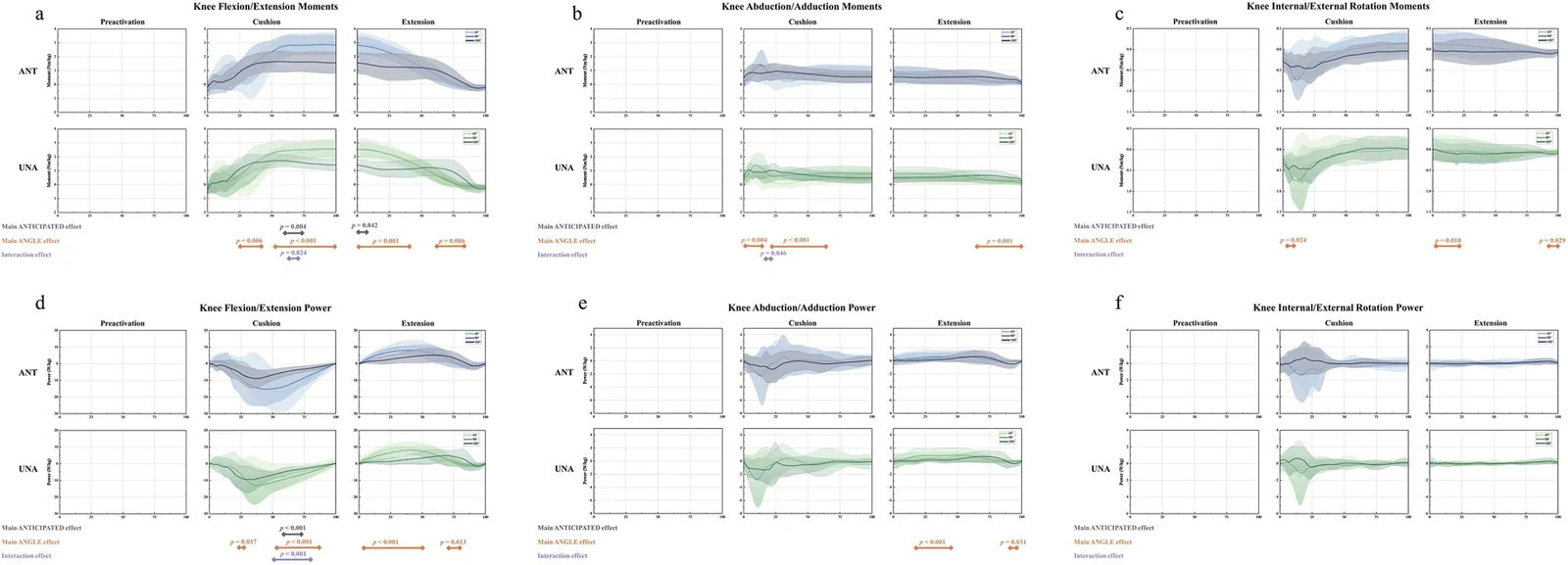
Knee moments and power profiles. **(a)** Knee Flexion/Extension Moments; **(b)** Knee Abduction/Adduction Moments; **(c)** Knee Internal/External Rotation Moments; **(d)** Knee Flexion/Extension Power; **(e)** Knee Abduction/Adduction Power; **(f)** Knee Internal/External Rotation Power. Data are presented for Anticipated (ANT, blue) and Unanticipated (UNA, green) conditions at 45°, 90°, and 180° cutting angles. Solid lines and shaded areas represent the mean and SD, respectively. Phases (Preactivation, Cushion, Extension) and horizontal bars indicating significant effects (SPM1D, *p* < 0.05) are defined as in Figure 1. Positive values indicate knee extension, abduction, internal rotation.

As illustrated in Figure 2a, Knee flexion/extension moments exhibited significant anticipation (60.2%*–*75.9%, *p =* 0.004), angle (25.4%*–*39.8%, *p =* 0.006; 50.1%*–*100.0%, *p <* 0.001), and interaction effects (62.1%*–*70.6%, *p =* 0.024) during the cushion phase, with anticipation (0.5%*–*6.9%, *p =* 0.042) and angle effects (0.0%*–*39.7%, *p <* 0.001; 62.4%*–*84.3%, *p =* 0.006) remaining significant during extension. Knee abduction/adduction moments showed significant angle effects during cushion (0.0%*–*13.5%, *p =* 0.004; 21.0%*–*63.8%, *p <* 0.001), and late extension (63.4%*–*100.0%, *p =* 0.001), with a significant interaction during early cushion (19.9%*–*22.2%, *p =* 0.046) as shown in Figure 2b. Knee rotation moments revealed significant angle effects during early cushion (5.5%*–*11.8%, *p =* 0.024), and extension (4.0%*–*23.3%, *p =* 0.010; 88.8%*–*100.0%, *p =* 0.029) as shown in Figure 2c. Knee flexion/extension power was significantly influenced during cushion by anticipation (54.2%*–*74.4%, *p <* 0.001), angle (24.6%*–*28.6%, *p =* 0.037; 54.6%*–*87.3%, *p <* 0.001), and interaction (49.6%*–*82.4%, *p <* 0.001), with angle effects persisting through extension (3.1%*–*51.5%, *p <* 0.001; 72.5%*–*83.4%, *p =* 0.013) as shown in Figure 2d. Knee abduction/adduction power showed significant angle effects during extension (19.0%*–*46.8%, *p <* 0.001; 91.7%*–*97.6%, *p =* 0.031) as shown in Figure 2e. No significant differences were observed in knee internal/external rotation power (Figure 2f).

Post-hoc analysis (Table 3) identified significant anticipation effects on CoM velocity at 45° (all phases, *p* < 0.001) and 90° (preactivation and cushion, *p* = 0.003). At 45°, ANT and UNA differed significantly in vertical GRF (cushion), knee power (cushion), and knee flexion angles (late preactivation and cushion) (*p* = 0.013). Knee moments exhibited extensive anticipation effects at 45° and 90° across sagittal and non-sagittal planes (*p* = 0.014). For the angle factor, increasing from 45° to 180° progressively decreased CoM velocity and vertical GRF (*p* < 0.001). Compared to larger angles, the 45° condition showed significantly higher knee flexion, power, and peak sagittal/frontal moments (*p* < 0.001; Supplementary Table A2).

**TABLE 3:** Post-hoc variables comparisons between anticipated and unanticipated conditions (N = 23).

Variables	Angle	Phase	Cluster (%)	Peak *t*-value	*p-value*	*Effect Size*
Resultant CoM velocity (m/s)	45°	PRE	0.0–99.0	6.495	<0.001	1.575
		CSH	0.0–99.0	5.450	<0.001	1.322
		EXT	0.0–99.0	4.398	0.001	1.067
	90°	PRE	0.0–99.0	3.823	0.003	0.927
		CSH	19.0–48.6	3.844	0.003	0.932
Vertical GRF (N/kg)	45°	CSH	19.1–24.3	4.203	0.013	1.019
Knee flex/Extension angle (°)	45°	PRE	82.4–99.0	5.090	0.010	1.234
		CSH	0.0–41.2	5.019	0.005	1.217
Knee flex/Extension moments (nm/kg)	45°	CSH	56.1–99.0	5.942	<0.001	1.441
		EXT	0.0–18.1	5.115	0.002	1.241
	90°	CSH	61.1–81.2	5.079	<0.001	1.232
Knee add/Abduction moments (nm/kg)	45°	CSH	14.4–17.6	4.402	0.014	1.068
Knee inter/External rotation moments (nm/kg)	90°	CSH	69.6–88.1	5.748	<0.001	1.394
Knee flex/Extension power (W/kg)	45°	CSH	54.4–75.4	−4.571	<0.001	−1.109

PRE, preactivation phase; CSH, cushion phase; EXT, extension phase. Positive/negative *t*-values denote ANT > UNA, and ANT < UNA, respectively. Significant Cluster (%) is the duration exceeding the critical threshold (*p* < 0.05, Bonferroni-corrected). Effect sizes (*d*): 0.2 (small), 0.5 (medium), and 0.8 (large).

## 4 Discussion

Our primary biomechanical findings indicate that the 45° UNA condition represents a biomechanically demanding movement condition characterized by altered dynamic joint mechanics, rather than direct evidence of biological tissue damage. This underscores a pronounced “performance-safety trade-off” uniquely exacerbated during reactive, small-angle cutting (Alimoradi et al., 2024; Zago et al., 2024). Central to this risk is the “angle-velocity trade-off” (Dos’Santos et al., 2018): unlike 90° or 180° maneuvers where trajectory constraints necessitate deceleration, the 45° task allows greater horizontal momentum to be redirected during rapid reorientation. From a motor control perspective, when unanticipated directional changes disrupt anticipated motor programs, this preserved momentum must be redirected within a compressed control loop, transforming the established movement momentum into elevated multi-planar mechanical demands (Brown TN et al., 2014).

The cutting angle fundamentally dictates the movement’s sensitivity to anticipation via the available temporal buffer (Jones et al., 2016). In 180° maneuvers, the extended timeframe provides sufficient window for anticipatory postural adjustments and systemic neuromuscular reorganization. As a secondary observation, the lack of significant changes in median frequency (MDF) across conditions suggests that the observed movement adaptations were unlikely to be primarily driven by peripheral muscle fatigue. In contrast, the 45° UNA task necessitates processing extreme inertial loads within a severely compressed window, leaving insufficient time for real-time sensorimotor corrections prior to peak joint loading (Brown SR et al., 2014; Bencke and Zebis, 2011; Zago et al., 2024). This extreme time constraint may challenge feed-forward neuromuscular regulation, manifesting as a pronounced postural lag—specifically delayed trunk flexion and axial rotation during preactivation (Brown SR et al., 2014; Besier et al., 2001). Consequently, this altered spatial orientation may increase mechanical demands on the joint system, potentially through an expanded external moment arm of the ground reaction force vector relative to the knee joint center (Landry et al., 2007).

In evaluating energy attenuation, absolute impact force magnitudes alone do not dictate joint safety; indeed, a lower peak vGRF under 45° UNA does not inherently represent a protective motor strategy. Rather, the interaction between vGRF, kinematic alignment, and power absorption reveals the underlying attenuation mechanics. While anticipated maneuvers utilize “pre-braking” strategies to modulate limb stiffness and safely attenuate ground forces (Liew et al., 2021), the UNA condition lacks this pre-planned motor program. Consequently, the knee joint exhibits a distinct surge in negative power during the early cushion phase. This negative power surge signifies an elevated rate of eccentric work imposed on the knee extensors to absorb kinetic energy within a restricted range of motion. Without a proactive muscular buffer to coordinate trunk orientation and knee flexion kinematics simultaneously, the lower extremity is forced into a reactive eccentric absorption strategy, suggesting altered mechanical demand distribution placed upon both active musculo-tendinous units and surrounding passive constraints (Maniar et al., 2019; Maniar et al., 2020).

Consistent with our primary hypothesis, the interaction between anticipation and cutting angle revealed phase-specific alterations in neuromuscular control and joint mechanics. At the local level, analyzing Condition × Angle interaction effects unveils critical neuromuscular mechanisms that cannot be captured by main effects alone. At 180°, the neuromuscular system demonstrates task-specific plasticity by actively modulating muscle activation (e.g., condition-dependent shifts in MG preactivation and VM cushion activation) to accommodate uncertainty (Laddawong et al., 2024). Conversely, at 45° UNA, the absence of such adaptive recruitment scaling highlights a collapse in real-time neuromuscular flexibility. This forces reliance on generalized muscle co-contraction strategies that struggle to absorb high entry forces dynamically (Besier et al., 2001). Concurrently, this sagittal-plane demand is compounded in the frontal plane, where peak KAM act on a joint influenced by postural lag. Crucially, KAM represents a net internal joint moment calculated via inverse dynamics—reflecting global multi-planar mechanical demand and lateral shear control strategies rather than direct measurement of ACL tissue loading (Brown SR et al., 2014). Indeed, accurately quantifying ACL loading requires integrating broader dynamic movement patterns and tissue-level modeling approaches; therefore, the present biomechanical patterns should be interpreted as indicators of ACL-related mechanical demands rather than direct measures of ligament loading (Xu et al., 2023). Within a motor control and neuro-mechanical plasticity framework (Sun et al., 2021), 45° UNA exemplifies a limit of central motor adaptability, where the convergence of pre-programmed postural deficits and constrained mechanical adjustments increases overall joint vulnerability, aligning our laboratory observations with clinical injury risk profiles.

### 4.1 Practical applications

Beyond characterizing movement demands, these findings may provide a framework for athletic conditioning and biomechanical assessment. Because the 45° UNA maneuver uniquely stresses the motor control system by combining high approach velocity with a severely restricted preparation timeframe, this task paradigm may provide a challenging reactive condition for examining neuromuscular control strategies under constrained preparation time. Recent consensus highlights that standard, pre-planned tasks may not fully capture reactive movement demands encountered during sport-specific situations due to a lack of reactive, match-like constraints (Buckthorpe, 2019; Webster and Hewett, 2019). This paradigm may be considered when examining neuromuscular control strategies under reactive constraints during advanced athletic assessment. Furthermore, the observed alterations in feedforward neuromuscular regulation and eccentric energy absorption may warrant further investigation regarding their potential relevance for neuromuscular training strategies. Previous intervention studies have suggested that multi-directional hop-stabilization and dynamic balance exercises may influence pre-activation patterns and dynamic joint control; however, whether such interventions can modify responses during reactive cutting maneuvers requires further investigation (Buckthorpe, 2019).

## 5 Limitation

Despite the meticulous experimental control—characterized by a randomized, counterbalanced design, strict approach velocity gating, and high-frequency synchronized EMG recording—certain targeted limitations warrant consideration. First, our cohort was restricted to high-level male soccer players (Chinese National First- and Second-Grade soccer players); therefore, these findings cannot be directly generalized to female players or athletes of different competitive levels. Additionally, despite multiplicity control within each statistical model, the number of complementary variables may increase the risk of type-I error inflation, and these exploratory findings should be interpreted cautiously. Second, while the custom LED-triggering system was designed to reduce available preparation time, it cannot fully reproduce the perceptual and temporal complexity of reactive situations encountered during match play. The electronic latency of the system and trial-specific cue-to-contact intervals were not independently quantified, which should be considered when interpreting the unanticipated condition. Furthermore, testing was limited to the dominant limb, which may not fully represent potential neuromuscular differences between limbs. Additionally, the non-fatigued laboratory setting with standardized indoor surfaces and footwear may not fully replicate the cleat-turf interactions and fatigue-related demands of competitive play. Finally, the standardized preactivation window may not fully capture individual variations in neuromuscular timing across different cutting angles. Lastly, future investigations integrating musculoskeletal modeling and advanced approaches are needed to clarify how biomechanical indicators relate to tissue-level loading mechanisms (Xu et al., 2025).

## 6 Conclusion

In conclusion, 45° UNA maneuvers represent a high-demand movement condition that exposes latent lower-limb biomechanical vulnerabilities under restricted preparation time. The combination of high approach momentum and limited planning opportunity may compromise anticipatory neuromuscular regulation, resulting in greater reliance on reactive energy absorption during dynamic loading. Compared with larger-angle maneuvers (90° and 180°), the 45° UNA task provides a narrower control window and places greater demands on movement regulation. These findings indicate that 45° UNA maneuvers may represent a challenging biomechanical paradigm for examining neuromuscular control strategies and biomechanical characteristics associated with ACL injury mechanisms.

## Data Availability

The raw data supporting the conclusions of this article will be made available by the authors, without undue reservation.
