# Supplementary material for: Anticipation-angle interaction identifies 45° unanticipated change of direction as a high-demand movement with altered ACL injury-related biomechanical characteristics
Source: Front Bioeng Biotechnol. 2026 Sep 9;14:1895455. doi: 10.3389/fbioe.2026.1895455 (PMC13597675; doi:10.3389/fbioe.2026.1895455)
Supplement: Supplementary file 1 [file Table1.docx]

Supplementary Material

# 1 Supplementary Tables

**Table A1.** Supplementary analysis of lower limb muscle Median Frequency (MDF) (*N* = 23)

| **Muscle** | **Phase** | **Con.** | **Angle (Mean ± SD)** | | | **ANOVA** | | | | | |  |
| --- | --- | --- | --- | --- | --- | --- | --- | --- | --- | --- | --- | --- |
|  |  |  |  |  |  | **Anticipation** | | **Angle** | | **Interaction** | | |
|  |  |  | **45°** | **90°** | **180°** | ***p*** | $\boldsymbol{\eta}_{\boldsymbol{p}}^{\boldsymbol{2}}$ | ***p*** | $\boldsymbol{\eta}_{\boldsymbol{p}}^{\boldsymbol{2}}$ | ***p*** | $\boldsymbol{\eta}_{\boldsymbol{p}}^{\boldsymbol{2}}$ | |
| Gastrocnemius Medialis (Hz) | PRE | ANT | 118.46±32.36 | 115.34±42.11 | 113.22±39.78 | 0.512 | 0.018 | 0.885 | 0.001 | 0.392 | 0.029 | |
|  |  | UNA | 112.44±38.46 | 119.78±36.67 | 114.54±40.21 |  |  |  |  |  |  | |
|  | CSH | ANT | 125.11±38.41 | 128.42±44.21 | 123.44±42.11 | 0.352 | 0.032 | 0.785 | 0.003 | 0.315 | 0.038 | |
|  |  | UNA | 121.22±41.54 | 131.54±40.81 | 126.88±41.22 |  |  |  |  |  |  | |
|  | EXT | ANT | 108.44±35.11 | 111.22±39.44 | 107.55±38.11 | 0.421 | 0.025 | 0.912 | 0.001 | 0.455 | 0.022 | |
|  |  | UNA | 105.11±37.88 | 114.33±36.55 | 110.22±39.44 |  |  |  |  |  |  | |
| Gastrocnemius Lateralis (Hz) | PRE | ANT | 115.22±39.11 | 118.44±41.22 | 114.11±40.55 | 0.388 | 0.028 | 0.843 | 0.001 | 0.372 | 0.030 | |
|  |  | UNA | 111.44±40.22 | 122.11±38.44 | 117.55±39.11 |  |  |  |  |  |  | |
|  | CSH | ANT | 110.11±34.55 | 113.22±37.11 | 109.44±36.88 | 0.442 | 0.023 | 0.875 | 0.001 | 0.412 | 0.026 | |
|  |  | UNA | 107.44±36.11 | 116.55±35.44 | 112.11±38.11 |  |  |  |  |  |  | |
|  | EXT | ANT | 113.55±38.11 | 116.44±40.22 | 112.11±39.55 | 0.395 | 0.027 | 0.822 | 0.002 | 0.384 | 0.029 | |
|  |  | UNA | 109.22±39.44 | 120.33±37.88 | 115.44±40.11 |  |  |  |  |  |  | |
| Vastus Medialis (Hz) | PRE | ANT | 117.22±40.11 | 120.44±43.11 | 116.11±41.55 | 0.368 | 0.031 | 0.795 | 0.003 | 0.334 | 0.036 | |
|  |  | UNA | 113.55±42.44 | 124.22±41.22 | 119.44±42.88 |  |  |  |  |  |  | |
|  | CSH | ANT | 117.44±35.11 | 114.22±40.55 | 112.11±41.22 | 0.485 | 0.020 | 0.843 | 0.001 | 0.421 | 0.026 | |
|  |  | UNA | 111.78±39.22 | 118.44±38.11 | 113.88±38.46 |  |  |  |  |  |  | |
|  | EXT | ANT | 123.78±37.11 | 126.88±42.78 | 122.11±40.55 | 0.375 | 0.030 | 0.752 | 0.004 | 0.338 | 0.035 | |
|  |  | UNA | 119.54±40.21 | 129.22±39.46 | 124.54±40.11 |  |  |  |  |  |  | |
| Vastus Lateralis (Hz) | PRE | ANT | 107.11±33.44 | 110.44±38.11 | 106.55±37.22 | 0.445 | 0.022 | 0.892 | 0.001 | 0.472 | 0.020 | |
|  |  | UNA | 104.22±36.55 | 113.11±35.11 | 109.44±38.11 |  |  |  |  |  |  | |
|  | CSH | ANT | 114.22±38.11 | 117.11±40.22 | 113.44±39.44 | 0.412 | 0.025 | 0.812 | 0.002 | 0.405 | 0.027 | |
|  |  | UNA | 110.11±39.44 | 121.22±37.11 | 116.55±38.11 |  |  |  |  |  |  | |
|  | EXT | ANT | 109.11±32.44 | 112.44±36.11 | 108.55±35.11 | 0.465 | 0.021 | 0.852 | 0.001 | 0.434 | 0.024 | |
|  |  | UNA | 106.44±35.11 | 115.11±34.55 | 111.44±37.44 |  |  |  |  |  |  | |
| Rectus Femoris (Hz) | PRE | ANT | 112.11±37.11 | 115.44±39.44 | 111.55±38.11 | 0.422 | 0.024 | 0.792 | 0.003 | 0.415 | 0.026 | |
|  |  | UNA | 108.44±38.55 | 119.11±36.44 | 114.22±39.11 |  |  |  |  |  |  | |
|  | CSH | ANT | 116.11±39.44 | 119.22±42.11 | 115.44±40.22 | 0.385 | 0.029 | 0.772 | 0.003 | 0.365 | 0.032 | |
|  |  | UNA | 112.44±41.11 | 123.11±40.11 | 118.55±41.44 |  |  |  |  |  |  | |
|  | EXT | ANT | 116.78±34.22 | 115.88±39.11 | 111.44±38.46 | 0.452 | 0.023 | 0.812 | 0.002 | 0.388 | 0.030 | |
|  |  | UNA | 110.54±37.78 | 117.22±35.67 | 112.78±36.11 |  |  |  |  |  |  | |
| Biceps Femoris (Hz) | PRE | ANT | 122.54±36.22 | 125.22±41.54 | 121.22±39.22 | 0.398 | 0.028 | 0.721 | 0.005 | 0.362 | 0.032 | |
|  |  | UNA | 118.22±39.11 | 127.78±38.22 | 123.54±39.46 |  |  |  |  |  |  | |
|  | CSH | ANT | 106.22±32.11 | 109.11±37.44 | 105.78±36.22 | 0.468 | 0.020 | 0.862 | 0.001 | 0.495 | 0.018 | |
|  |  | UNA | 103.44±35.44 | 112.44±34.22 | 108.11±37.11 |  |  |  |  |  |  | |
|  | EXT | ANT | 113.11±37.44 | 116.44±39.11 | 112.78±38.11 | 0.434 | 0.024 | 0.782 | 0.003 | 0.421 | 0.026 | |
|  |  | UNA | 109.22±38.11 | 120.11±36.44 | 115.44±37.11 |  |  |  |  |  |  | |
| Semitendinosus (Hz) | PRE | ANT | 108.22±31.55 | 111.44±35.22 | 107.44±34.44 | 0.482 | 0.019 | 0.832 | 0.001 | 0.455 | 0.022 | |
|  |  | UNA | 105.11±34.11 | 114.22±33.11 | 110.78±36.11 |  |  |  |  |  |  | |
|  | CSH | ANT | 111.44±36.11 | 114.22±38.11 | 110.11±37.44 | 0.445 | 0.022 | 0.765 | 0.004 | 0.432 | 0.025 | |
|  |  | UNA | 107.22±37.55 | 118.44±35.22 | 113.44±38.11 |  |  |  |  |  |  | |
|  | EXT | ANT | 115.11±38.11 | 118.44±41.22 | 114.44±39.11 | 0.402 | 0.027 | 0.742 | 0.004 | 0.385 | 0.029 | |
|  |  | UNA | 111.44±40.22 | 122.11±39.11 | 117.11±40.44 |  |  |  |  |  |  | |

Note: ANT, anticipated condition; UNA, unanticipated condition; Con., condition. PRE, preactivation; CSH, cushion; EXT, extension. For all Two-way Repeated Measures ANOVA effects, the degrees of freedom are *df*(1, 22) for the main effect of Condition, *df*(2, 44) for the main effect of Angle, and *df*(2, 44) for the Interaction effect. Superscript letters denote significant pairwise comparisons between angles: a (45° vs. 90°), b (90° vs. 180°), and c (45° vs. 180°). The symbol $ denotes a significant simple main effect between ANT and UNA conditions at the same angle. Single symbols (e.g., a, $) represent *p* < 0.05, while double symbols (e.g., aa, $$) represent *p* < 0.01.

**Table A2.** SPM1D post-hoc statistical cluster summary for lower limb biomechanics (*N* = 23)

| **Variable** | **Phase** | **Con.** | **Angle**  **Comparison** | **Significant Cluster (%)** | **Peak**  ***t-value*** | ***p-value*** | **Effect Size** |
| --- | --- | --- | --- | --- | --- | --- | --- |
| Resultant CoM Velocity (m/s) | PRE | ANT | 45° vs 90° | 0.0 – 99.0 | 12.919 | <0.001 | 3.133 |
|  |  |  | 45° vs 180° | 0.0 – 99.0 | 17.468 | <0.001 | 4.237 |
|  |  |  | 90° vs 180° | 0.0 – 99.0 | 13.268 | <0.001 | 3.218 |
|  |  | UNA | 45° vs 90° | 5.8 – 99.0 | 6.836 | 0.003 | 1.658 |
|  |  |  | 45° vs 180° | 0.0 – 99.0 | 11.480 | <0.001 | 2.784 |
|  |  |  | 90° vs 180° | 6.4 – 99.0 | 6.671 | 0.001 | 1.618 |
|  | CSH | ANT | 45° vs 90° | 0.0 – 99.0 | 14.736 | <0.001 | 3.574 |
|  |  |  | 45° vs 180° | 0.0 – 99.0 | 25.204 | <0.001 | 6.113 |
|  |  |  | 90° vs 180° | 0.0 – 99.0 | 14.730 | <0.001 | 3.573 |
|  |  | UNA | 45° vs 90° | 0.0 – 99.0 | 14.189 | <0.001 | 3.441 |
|  |  |  | 45° vs 180° | 0.0 – 99.0 | 22.920 | <0.001 | 5.559 |
|  |  |  | 90° vs 180° | 0.0 – 99.0 | 14.595 | <0.001 | 3.540 |
|  | EXT | ANT | 45° vs 90° | 0.0 – 99.0 | 15.800 | <0.001 | 3.832 |
|  |  |  | 45° vs 180° | 0.0 – 99.0 | 30.004 | <0.001 | 7.277 |
|  |  |  | 90° vs 180° | 0.0 – 99.0 | 23.267 | <0.001 | 5.643 |
|  |  | UNA | 45° vs 90° | 0.0 – 99.0 | 17.554 | <0.001 | 4.258 |
|  |  |  | 45° vs 180° | 0.0 – 99.0 | 40.455 | <0.001 | 9.812 |
|  |  |  | 90° vs 180° | 0.0 – 99.0 | 26.616 | <0.001 | 6.455 |
| Vertical GRF (N/kg) | CSH | ANT | 45° vs 180° | 25.8 – 99.0 | 10.988 | <0.001 | 2.665 |
|  |  |  | 90° vs 180° | 8.5 – 15.1 | 6.375 | 0.003 | 1.546 |
|  |  |  |  | 50.8 – 99.0 | 10.813 | <0.001 | 2.623 |
|  |  | UNA | 45° vs 180° | 43.0 – 99.0 | 8.523 | <0.001 | 2.067 |
|  |  |  | 90° vs 180° | 49.7 – 99.0 | 7.536 | <0.001 | 1.828 |
|  | EXT | ANT | 45° vs 90° | 18.5 – 30.5 | 4.492 | <0.001 | 1.089 |
|  |  |  | 45° vs 180° | 0.0 – 48.8 | 12.974 | <0.001 | 3.147 |
|  |  |  |  | 68.9 – 83.3 | -5.120 | <0.001 | -1.242 |
|  |  |  | 90° vs 180° | 0.0 – 47.2 | 13.075 | <0.001 | 3.171 |
|  |  | UNA | 45° vs 180° | 0.0 – 47.4 | 10.202 | <0.001 | 2.474 |
|  |  |  |  | 68.0 – 98.3 | -6.160 | <0.001 | -1.494 |
|  |  |  | 90° vs 180° | 0.0 – 43.5 | 9.606 | <0.001 | 2.330 |
|  |  |  |  | 68.4 – 98.6 | -7.648 | <0.001 | -1.855 |
| Knee Flexion/Extension Angle (°) | PRE | ANT | 45° vs 90° | 75.2 – 99.0 | -5.109 | 0.003 | -1.239 |
|  |  | UNA | 45° vs 90° | 70.5 – 99.0 | -10.743 | 0.002 | -2.605 |
|  |  |  | 45° vs 180° | 58.3 – 99.0 | -12.448 | <0.001 | -3.019 |
|  |  |  | 90° vs 180° | 74.3 – 99.0 | -4.623 | 0.002 | -1.121 |
|  | CSH | ANT | 45° vs 90° | 0.0 – 19.7 | -5.190 | 0.004 | -1.259 |
|  |  |  | 45° vs 180° | 50.3 – 99.0 | 3.939 | 0.006 | 0.955 |
|  |  | UNA | 45° vs 90° | 0.0 – 23.8 | -11.046 | 0.005 | -2.679 |
|  |  |  | 45° vs 180° | 0.0 – 20.1 | -11.453 | 0.005 | -2.778 |
|  |  |  | 90° vs 180° | 0.0 – 5.4 | -4.579 | 0.008 | -1.111 |
|  | EXT | ANT | 45° vs 180° | 0.0 – 63.3 | 4.672 | <0.001 | 1.133 |
|  |  |  | 90° vs 180° | 25.6 – 46.7 | 3.793 | 0.006 | 0.920 |
|  |  | UNA | 45° vs 180° | 30.6 – 77.6 | 5.419 | <0.001 | 1.314 |
|  |  |  | 90° vs 180° | 41.6 – 77.6 | 5.991 | <0.001 | 1.453 |
|  | CSH | ANT | 45° vs 180° | 74.2 – 99.0 | 4.394 | <0.001 | 1.066 |
|  |  | UNA | 45° vs 180° | 65.3 – 99.0 | 6.089 | <0.001 | 1.477 |
|  |  |  | 90° vs 180° | 77.4 – 91.3 | 4.326 | 0.002 | 1.049 |
| Knee Internal/External Rotation Angle (°) | EXT | ANT | 45° vs 90° | 0.0 – 13.4 | 4.151 | 0.005 | 1.007 |
|  |  |  | 45° vs 180° | 0.0 – 12.8 | 4.443 | 0.005 | 1.078 |
|  |  |  |  | 25.2 – 58.9 | 4.208 | <0.001 | 1.021 |
|  |  | UNA | 45° vs 180° | 0.0 – 72.5 | 5.431 | <0.001 | 1.317 |
|  |  |  | 90° vs 180° | 18.5 – 68.6 | 5.056 | <0.001 | 1.226 |
| Knee Flexion/Extension Moments (Nm/kg) | CSH | ANT | 45° vs 180° | 58.3 – 99.0 | 5.959 | <0.001 | 1.445 |
|  |  |  | 90° vs 180° | 54.4 – 99.0 | 8.331 | <0.001 | 2.020 |
|  |  | UNA | 45° vs 180° | 22.5 – 34.1 | -5.387 | <0.001 | -1.307 |
|  |  |  |  | 73.9 – 99.0 | 5.806 | <0.001 | 1.408 |
|  |  |  | 90° vs 180° | 57.7 – 99.0 | 6.745 | <0.001 | 1.636 |
|  | EXT | ANT | 45° vs 180° | 0.0 – 20.2 | 5.738 | <0.001 | 1.392 |
|  |  |  | 90° vs 180° | 0.0 – 29.4 | 8.697 | <0.001 | 2.109 |
|  |  | UNA | 45° vs 180° | 0.0 – 28.8 | 6.188 | <0.001 | 1.501 |
|  |  |  | 90° vs 180° | 0.0 – 33.1 | 7.378 | <0.001 | 1.789 |
|  |  |  |  | 66.2 – 74.6 | -4.818 | 0.003 | -1.168 |
|  |  |  |  | 95.6 – 99.0 | 5.174 | 0.007 | 1.255 |
| Knee Adduction/Abduction Moments (Nm/kg) | CSH | ANT | 45° vs 90° | 27.8 – 46.1 | -8.090 | <0.001 | -1.962 |
|  |  |  | 45° vs 180° | 29.4 – 35.0 | -5.021 | 0.004 | -1.218 |
|  |  | UNA | 45° vs 90° | 5.5 – 10.7 | -5.357 | 0.004 | -1.299 |
|  |  |  |  | 23.1 – 41.9 | -6.189 | <0.001 | -1.501 |
|  |  |  | 45° vs 180° | 0.0 – 2.3 | -5.688 | 0.005 | -1.380 |
|  |  |  |  | 17.9 – 51.1 | -7.501 | <0.001 | -1.819 |
|  | EXT | UNA | 45° vs 90° | 98.6 – 99.0 | 3.784 | 0.008 | 0.918 |
|  |  |  | 45° vs 180° | 68.4 – 81.0 | -4.933 | <0.001 | -1.196 |
|  |  |  |  | 91.0 – 98.0 | -5.406 | 0.004 | -1.311 |
|  |  |  | 90° vs 180° | 72.7 – 99.0 | -8.689 | <0.001 | -2.107 |
| Knee Internal/External Rotation Moments (Nm/kg) | CSH | UNA | 45° vs 90° | 4.7 – 9.0 | 4.919 | 0.005 | 1.193 |
|  | EXT | UNA | 90° vs 180° | 90.4 – 99.0 | 5.287 | 0.002 | 1.282 |
| Knee Flexion/Extension Power (W/kg) | CSH | ANT | 90° vs 180° | 51.2 – 95.1 | -6.439 | <0.001 | -1.562 |
|  | EXT | ANT | 45° vs 180° | 6.1 – 38.4 | 8.472 | <0.001 | 2.055 |
|  |  |  | 90° vs 180° | 2.5 – 39.2 | 6.874 | <0.001 | 1.667 |
|  |  | UNA | 45° vs 180° | 4.8 – 44.7 | 7.836 | <0.001 | 1.901 |
|  |  |  | 90° vs 180° | 6.6 – 13.3 | 4.673 | 0.002 | 1.133 |
|  |  |  |  | 19.2 – 45.5 | 6.467 | <0.001 | 1.568 |
| Trunk Flexion Angle (°) | PRE | ANT | 45° vs 90° | 26.3 – 87.8 | 4.578 | <0.001 | 1.110 |
|  |  |  | 45° vs 180° | 0.0 – 99.0 | 6.844 | <0.001 | 1.660 |
|  |  |  | 90° vs 180° | 1.2 – 99.0 | 4.474 | <0.001 | 1.085 |
|  |  | UNA | 45° vs 90° | 8.9 – 90.0 | 6.250 | <0.001 | 1.516 |
|  |  |  | 45° vs 180° | 0.0 – 99.0 | 9.756 | <0.001 | 2.366 |
|  |  |  | 90° vs 180° | 5.9 – 99.0 | 5.813 | <0.001 | 1.410 |
|  | CSH | ANT | 45° vs 90° | 50.9 – 99.0 | 4.124 | 0.005 | 1.000 |
|  |  |  | 45° vs 180° | 0.0 – 99.0 | 5.496 | <0.001 | 1.333 |
|  |  |  | 90° vs 180° | 0.0 – 99.0 | 4.120 | <0.001 | 0.999 |
|  |  | UNA | 45° vs 90° | 47.0 – 99.0 | 4.123 | 0.005 | 1.000 |
|  |  |  | 45° vs 180° | 0.0 – 99.0 | 6.981 | <0.001 | 1.693 |
|  |  |  | 90° vs 180° | 0.0 – 99.0 | 6.175 | <0.001 | 1.498 |
|  | EXT | ANT | 45° vs 90° | 0.0 – 99.0 | 5.241 | <0.001 | 1.271 |
|  |  |  | 45° vs 180° | 0.0 – 99.0 | 7.193 | <0.001 | 1.745 |
|  |  |  | 90° vs 180° | 0.0 – 81.8 | 5.147 | <0.001 | 1.248 |
|  |  | UNA | 45° vs 90° | 0.0 – 86.2 | 5.676 | <0.001 | 1.377 |
|  |  |  | 45° vs 180° | 0.0 – 95.1 | 7.421 | <0.001 | 1.800 |
|  |  |  | 90° vs 180° | 0.0 – 84.8 | 5.965 | <0.001 | 1.447 |
| Trunk Lateral Flexion Angle (°) | PRE | ANT | 45° vs 180° | 0.0 – 63.1 | 3.881 | <0.001 | 0.941 |
|  |  |  | 90° vs 180° | 0.0 – 72.7 | 5.225 | <0.001 | 1.267 |
|  |  | UNA | 90° vs 180° | 0.0 – 82.4 | 7.422 | <0.001 | 1.800 |
|  | EXT | UNA | 45° vs 90° | 87.5 – 99.0 | -4.131 | 0.008 | -1.002 |
|  |  |  | 45° vs 180° | 94.7 – 99.0 | -3.754 | 0.008 | -0.910 |
| Trunk Axial Rotation Angle (°) | PRE | ANT | 45° vs 90° | 41.7 – 55.8 | -4.056 | 0.006 | -0.984 |
|  |  | UNA | 45° vs 90° | 0.0 – 57.4 | -4.700 | <0.001 | -1.140 |
|  | CSH | UNA | 45° vs 180° | 91.3 – 99.0 | 3.843 | 0.008 | 0.932 |
|  | EXT | ANT | 45° vs 180° | 5.2 – 75.9 | 5.716 | <0.001 | 1.386 |
|  |  | UNA | 45° vs 180° | 0.0 – 75.5 | 6.055 | <0.001 | 1.469 |

Note: ANT, anticipated condition; UNA, unanticipated condition; Con., condition. PRE, preactivation; CSH, cushion; EXT, extension. Positive *t-values* indicate that the second-listed condition in the comparison (e.g., 180° in "45° vs. 180°") had a higher magnitude than the first-listed condition; negative values indicate the opposite. Significant Cluster (%) represents the specific duration within the movement phase where the SPM{t} curve exceeded the critical threshold (*p* < 0.008, Bonferroni-corrected for the angle effect). Effect sizes are reported as *Cohen's d*, where *d* > 0.2, 0.5, and 0.8 represent small, medium, and large effects, respectively.
